# Oxidative stress-mediated intrinsic apoptosis in human promyelocytic leukemia HL-60 cells induced by organic arsenicals

**DOI:** 10.1038/srep29865

**Published:** 2016-07-19

**Authors:** Xiao-Yang Fan, Xin-You Chen, Yu-Jiao Liu, Hui-Min Zhong, Feng-Lei Jiang, Yi Liu

**Affiliations:** 1 State Key Laboratory of Virology, College of Chemistry and Molecular Sciences, Wuhan University, Wuhan 430072, P. R. China; 2 School of Chemistry and Materials Science, Hubei Engineering University, Xiaogan 432000, P. R. China

## Abstract

Arsenic trioxide has shown the excellent therapeutic efficiency for acute promyelocytic leukemia. Nowadays, more and more research focuses on the design of the arsenic drugs, especially organic arsenicals, and on the mechanism of the inducing cell death. Here we have synthesized some organic arsenicals with Schiff base structure, which showed a better antitumor activity for three different kinds of cancer cell lines, namely HL-60, SGC 7901 and MCF-7. Compound **2a** (2-(((4-(oxoarsanyl)phenyl)imino)methyl)phenol) and **2b** (2-methoxy-4-(((4-(oxoarsanyl)phenyl)imino)methyl)phenol) were chosen for further mechanism study due to their best inhibitory activities for HL-60 cells, of which the half inhibitory concentration (*IC*_50_) were 0.77 μM and 0.51 μM, respectively. It was illustrated that **2a** or **2b** primarily induced the elevation of reactive oxygen species, decrease of glutathione level, collapse of mitochondrial membrane potential, release of cytochrome *c*, activation of Caspase-3 and apoptosis, whereas all of the phenomena can be eliminated by the addition of antioxidants. Therefore, we concluded that compound **2a** and **2b** can induce the oxidative stress-mediated intrinsic apoptosis in HL-60 cells. Both the simplicity of structure with Schiff base group and the better anticancer efficiency demonstrate that organic arsenicals are worthy of further exploration as a class of potent antitumor drugs.

Arsenic is a ubiquitous element in nature displaying some properties of both a metal and a nonmetal^1^,^2^. Environmental exposure to toxicants or metals can cause regulated and/or unregulated cell death resulting in tissue and cell injury^3^. Some studies find that chronic exposure to arsenic from groundwater and food could cause the largest environmental health disaster in the world and that is associated with the increased risk of a number of cancers^1^,^2^,^4^,^5^. Hence, arsenic is classified as a human carcinogen by the International Agency for Research on Cancer (IARC) and the U.S. Environmental Protection Agency (EPA)^1^.

Recently, arsenic trioxide (As_2_O_3_), which was used as a traditional Chinese medicine, has been reported to induce complete remission in the patients with acute promyelocytic leukemia (APL)^6^,^7^ and approved by the U.S. FDA in 2000^8^. Based on its remarkable clinical success, this drug has attracted wide interest for its pharmacological mechanisms. Many researches about the inhibition mechanism of arsenic trioxide for diverse cancer cells, including myeloma cells, malignant lymphocytes, mesothelioma cells, hepatoma cells, have been reported^9^,^10^,^11^,^12^. Actually, arsenical-based molecules have been used as therapeutic agents for centuries for various ailments such as psoriasis, syphilis, leukemia, and rheumatism^10^,^13^,^14^. In terms of chemistry, arsenic has two important oxidation states, As (III) and As (V), and the element is able to form both inorganic and organic compounds in the environment as well as within the human body. Inorganic arsenicals consist of an arsenic atom linked to other elements such as oxygen, sulfur and chlorine, whereas in combination with hydrogen and carbon, the element is considered as organic arsenic^2^. Compared with inorganic arsenicals, organic arsenicals have diverse structures and better bioavailability^15^. Arsphenamine, as a kind of organic arsenic agent, was used to treat syphilis and trypanosomiasis in the early 20th century^16^. Currently, more and more organic arsenicals are in process of investigation for cancer treatment.

Generally, programmed cell death is divided into apoptosis (PCDI), autophagy (PCDII), and necroptosis (PCDIII)^17^. Apoptosis is defined by a pattern of molecular and morphological changes that result in the packaging and removal of the dying cell^18^. Highly controlled by a range of complex signaling in cell, apoptosis is closely related to the tissue development, homeostasis and diseases^19^. There are two signaling pathways that triggering apoptosis: the extrinsic and the intrinsic pathways. The extrinsic apoptotic pathway is promoted by soluble molecules belonging to the Tumor Necrosis Factor (TNF) family, normally secreted as homotrimers, which can bind to plasma membrane receptors of the TNF-Receptor (TNFR) family, causing their trimerization and subsequent activation. The intrinsic apoptosis signaling pathway is associated with mitochondrial functions, including mtROS^20^,^21^. It has been reported that reactive oxygen species (ROS) play a vital role in DNA damage, cellular apoptosis, tissue damage and aging^22^,^23^,^24^. In addition, there is accumulating evidence that cancer cells’ response to apoptosis is regulated by the cellular redox status. For example, intracellular ROS have been shown to regulate hTERT at many levels, such as regulation of its expression and activity as well as modulating its localization and altering its functions^25^. ROS is also known to activate the mitogen activated protein kinase (MAPK) pathways which are important mediators of signal transduction and play a key role in regulating many cellular processes^26^. That many kinds of drugs can induce ROS-mediated apoptosis in a series of cancer cells has been reported in succession^15^,^27^,^28^.

As (III) can affect the conformation and function of numerous proteins through its high affinity for sulfhydryl groups, which results potential malfunctions in deoxyribonucleotide synthesis and repair, protein folding, sulfur metabolism and xenobiotic detoxification^1^,^24^. Reduced glutathione (GSH), the major cellular antioxidant, is likely to be oxidized by trivalent arsenicals, which leads to the increase of ROS^24^. In this case, a series of organic arsenicals have been synthesized (Fig. 1). Considering that Schiff bases have also been shown to exhibit a broad range of bioactivities, including antibacterial, antiproliferative and antiviral activities^29^, the synthesized compounds are a series of arsenic (III)-containing Schiff base group derivatives. These compounds all displayed the good inhibition rates for different kinds of cancer cell lines, especially HL-60 cells. The best inhibitory activity showed that using less than 1 μM compound (**2a** or **2b**) could kill the majority of HL-60 cells. By means of introducing a series of reductants we were able to reveal the apoptotic mechanism, which was mainly mediated by oxidative stress. In short, the addition of **2a** or **2b** induces the oxidative stress primarily, followed by the decrease of GSH content, collapse of mitochondrial membrane potential and the release of cytochrome *c*, resulting in the intrinsic apoptosis.

## Results

### Synthesis of organic arsenicals

**Figure 1:**
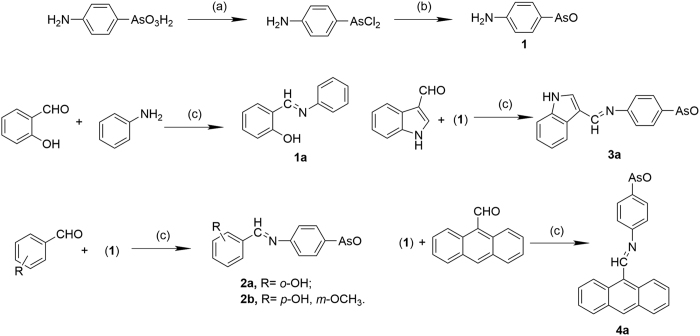
The synthesis of Schiff base derivatives. (**a**) concentrated hydrochloric acid and methanol (v/v: 24 mL/30 mL), catalytic amount of KI, SO_2_ (2 bubbles/s), RT, 30 min; (**b**) aqueous ammonia 10%, 0 °C, 15 min; (**c**) EtOH, 78–80 °C, 2–6 h, under N_2_.

The synthetic route of all compounds is illustrated in Fig. 1, including three steps (a, b and c) from *p*-Arsanilic acid (with no toxicity to human) to final products. Firstly, *p*-Arsanilic acid was reduced to form *p*-Aminophenylarsine oxide (**1**) via intermediate product *p*-aminophenylarsine dichloride. Secondly, the final products were synthesized by the condensation reaction of *p*-Aminophenylarsine oxide (**1**) with four aromatic aldehydes, namely salicylic aldehyde, vanillin, indole-3-mathanal and 9-anthraldehyde, generating compounds **2a**, **2b, 3a** and **4a** respectively. Additionally, the condensation reaction between salicylic aldehyde and aniline was occurred to yield the reference sample compound **1a**. The arsenicals must be treated with carefulness and proper safety procedures are indispensable.

### Assessment of cytotoxicity and compounds screening

The concentration of half inhibition of cell proliferation (*IC*_50_) is summarized in Table 1 and Supplementary Table S1. The inhibitions of these arsenicals for different cell lines all show a time and concentration dependent effect (data not shown). In details, compared with MCF-7, SGC7901 and GES-1 cells, compound **2a**, **2b**, **3a** and inorganic arsenicals display larger destruction potency for HL-60 cells. In contrast, the analogue compound **1a** without arsenic atom has almost no cytotoxicity and compound **4a** shows the more cytotoxicity for normal cells. In addition, among these arsenicals, **2a** and **2b** with *IC*_50_ of 0.77 μM and 0.51 μM respectively are the most effective compounds for HL-60 cells. Furthermore, compared with cancer cells, they are less toxic for normal cells, namely GES-1, HEK293 and NIH3T3 cells. Besides, similar with As_2_O_3_, **2a** and **2b** have no more influence on normal leukocyte than control (Supplementary Figures S1 and S2). In follow-up studies, we paid more attention on the influence of **2a** and **2b** on HL-60 cells.

### Induction of apoptosis

**Figure 2:**
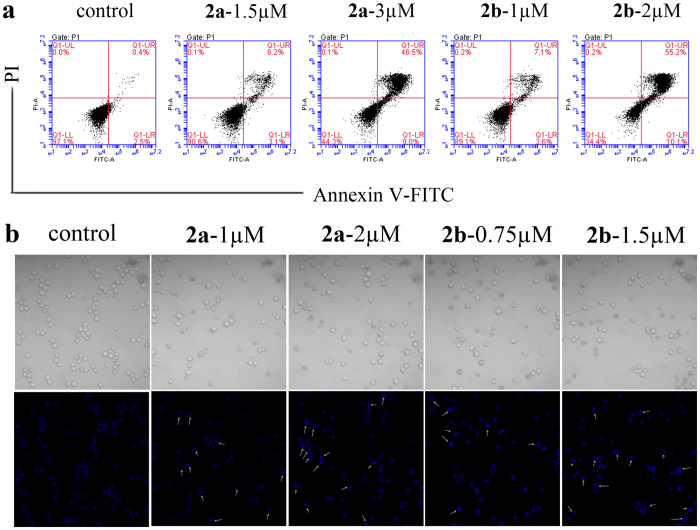
Induction of apoptosis in HL-60 cells *in vitro*. (**a**) The results of apoptosis by Annexin V-FITC/PI double-staining assay. HL-60 cells were treated with compound **2a** (1.5 μM and 3 μM) or **2b** (1 μM and 2 μM) for 24 h, then stained by Annexin V-FITC and PI dye. There are four kinds of cell populations shown as follows: live cells population (lower left), early apoptotic cells (lower right), late apoptotic cells (upper right) and dead cells (upper left). (**b**) Analysis of morphology changes in HL-60 cells. HL-60 cells were treated with **2a** (1 μM and 2 μM) or **2b** (0.75 μM and 1.5 μM) for 24 h followed by Hoechst 33342 staining. Phase contrast (top) and fluorescence (bottom) images were acquired by fluorescence microscopy. The apoptotic cells are shown with white arrows. These experiment were performed more than three times.

Arsenic compounds have been known to activate apoptosis signaling in numerous types of cell lines^30^,^31^,^32^,^33^. In our research, we also found **2a** and **2b** killed HL-60 cells mainly through triggering the induction of apoptosis, which was proved by the results shown in Fig. 2a. From Annexin V-FITC/PI double staining result, the apoptosis rates (FITC+/PI− and FITC+/PI+) were in proportion to the concentration of **2a** and **2b**. Specifically, the apoptosis rates of **2a** and **2b** were 9.3% (1.5 μM) and 55.5% (3 μM), 10.7% (1 μM) and 65.3% (2 μM), respectively, which were higher than that of control (2.9%). Whereas the percentages of necrotic cells (FITC−/PI+) in the presence or absence of **2a** or **2b** were as low as that of control ranging from 0.1% to 0.2%.

With the help of Hoechst 33342, the images (Fig. 2b) of HL-60 cells showed that the cell membrane became incomplete, cellular morphology changed and the number of cells decreased due to the addition of **2a** or **2b**. From the fluorescence figures, the cells with **2a** or **2b** displayed some high fluorescent fragments pointed by white arrows, characterizing cell nuclear shrinkage and DNA incision, which indicated that the cells were undergoing apoptosis. Meanwhile, these observations were not found in control sample.

### Production and elimination of ROS in HL-60 cells

**Figure 3:**
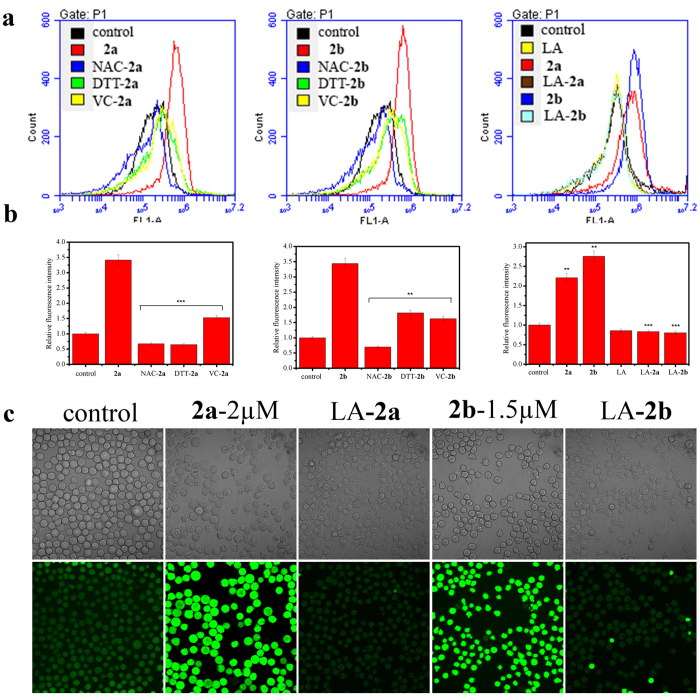
Production and elimination of ROS in HL-60 cells measured by DCFH-DA. (**a**) The levels of intracellular ROS in the presence of antioxidants and compound **2a** or **2b**. HL-60 cells were pre-incubated with 2 mM NAC or 1 mM DTT or 1 mM VC or 0.075 mM LA for 4 h followed by the addition of 2 μM **2a** or 1.5 μM **2b** for another 24 h and the fluorescence data were obtained by flow cytometry. (**b**) The corresponding quantification data are shown in the way of column figure and expressed as the mean ± SD of three independent samples. **P < 0.01 and ***P < 0.001 vs the control group. (**c**) The images about the accumulation and elimination of ROS in cells. HL-60 cells were treated with 0.075 mM LA in advance for 4 h before incubated with 2 μM **2a** or 1.5 μM **2b** for another 24 h. Then 1 mL FBS-free medium with 10 μM DCFH-DA was added into each sample for 30 min at 37 °C. Phase contrast (top) and fluorescence (bottom) images were acquired by fluorescence microscopy.

It has been demonstrated that arsenic compounds can increase the level of ROS in cells as a result of cell death^1^,^3^,^23^,^34^, so we assessed the change of ROS in HL-60 cells exposed to **2a** or **2b**. DCFH-DA, a kind of ROS probe, which is taken up by cells, hydrolyzed by cellular esterase to yield DCFH that in turn reacts with ROS to produce fluorescent DCF, was used in this assay^35^. According to the flow cytometry charts, the quantitative data were shown in the form of bar diagrams. With the respective addition of both compounds, the relative fluorescence intensity of DCF doubled approximately than that of control in Fig. 3a,b. However, we found that ROS can be eliminated by several general antioxidants, which were independent on the species of antioxidants. When the cells were incubated with compound **2a**, 2 mM NAC or 1 mM DTT can scavenge additional ROS completely, and 1 mM VC can also decrease the level of ROS to that of control. Moreover, 1 mM DTT or VC can eliminate the majority of ROS produced by compound **2b**, which can also be removed completely by 2 mM NAC. However, the addition of 0.075 mM LA was an effective approach for scavenging ROS completely produced by **2a** or **2b**. Besides, the change of intracellular ROS level can be displayed by a visual way with the help of fluorescent confocal microscope (Fig. 3c). The green light intensity characterizing ROS content became stronger owing to the addition of **2a** or **2b**, followed by weakening after pre-adding 0.075 mM LA, which were in accordance with the former results.

### Decrease in levels of total GSH in cells and the protective effect of GSH

**Figure 4:**
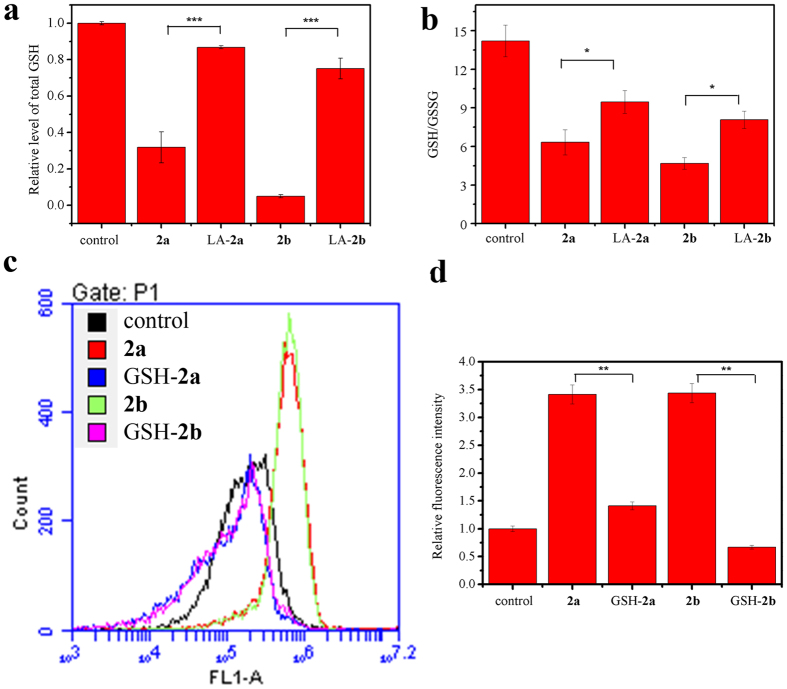
Assessment of total GSH in cells and the protective effect of GSH. (**a**) The total GSH level in cells under the treatment of 0.075 mM LA and 2 μM **2a** or 1.5 μM **2b**. The data are expressed as the mean ± SD of three independent samples. (**b**) The change of GSH/GSSH. The ratio was calculated according to the reduced GSH and GSSG levels. Data are expressed as the mean ± SD of three experiments. (**c**) The elimination effect of GSH for ROS. HL-60 cells were pre-incubated with 2 mM GSH for 4 h followed by adding 2 μM **2a** or 1.5 μM **2b** for another 24 h and the fluorescence data were obtained by flow cytometry. (**d**) The quantification data of fluorescence intensity are shown in the way of bar figure. Data are expressed as the mean ± SD of three experiments. *P < 0.05, **P < 0.01 and ***P < 0.001 vs the control group.

Because of GSH scavenging ROS in cells as described above, we speculated the change of GSH was contrary to that of ROS. As shown in Fig. 4, the content of intracellular total GSH decreased significantly when HL-60 cells exposed to **2a** or **2b**, whereas pretreatment of LA could recover the content. In addition, the ratio of GSH/GSSG showed the similar variation trend. In order to explore the role of GSH, we examined the protective effect of GSH. After being pretreated with GSH, the level of ROS in cells exposed to **2a** or **2b** began to decrease by a concentration-dependent way.

### Assessment of mitochondrial membrane potential and release of Cytochrome *c*

**Figure 5:**
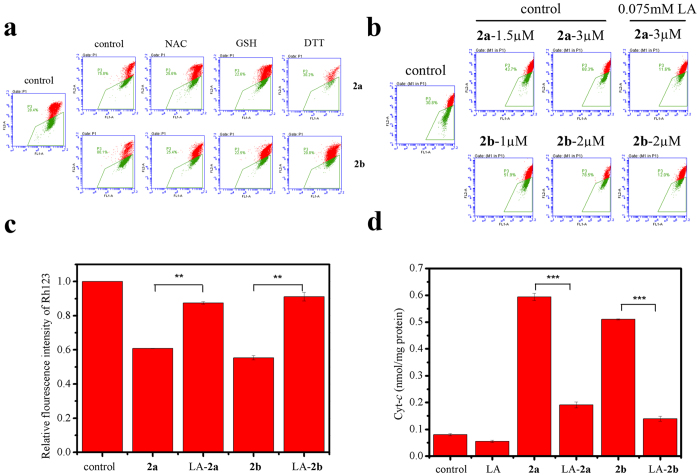
Assessment of mitochondrial membrane potential and release of cytochrome *c*. (**a**) The collapse and recovery of mitochondrial membrane potential. HL-60 cells were incubated with 3 μM **2a** or 2 μM **2b** in the presence or absence of 2 mM NAC, 2 mM GSH or 1 mM DTT for 24 h, followed by JC-1 staining. Data are obtained by the use of flow cytometry. (**b**) The alteration of mitochondrial membrane potential in HL-60 cells with or without 0.075 mM LA measured by JC-1 dye. This assay was repeated more than three times. (**c**) The change of mitochondrial membrane potential in cells monitored by Rh123. The cells were treated with 3 μM **2a** or 2 μM **2b** in the presence or absence of 0.075 mM LA. Data are recorded by flow cytometry and shown in the way of bar figure. The quantification data are expressed as the mean ± SD of three independent samples. **P < 0.01 vs the control group. (**d**) The release of cytochrome *c*. The cells were treated with 3 μM **2a** or 2 μM **2b** in the presence or absence of 0.075 mM LA. Data are expressed as the mean ± SD of three independent samples. ***P < 0.001 vs the control group.

When the level of GSH decreased, especially mitochondrial GSH, it can induce the loss of mitochondrial membrane potential^36^,^37^. Maintaining mitochondrial membrane potential is essential for keeping mitochondrial normal functions. As shown in Fig. 5b, the proportion of mitochondrial membrane potential collapse grew up significantly from 30.8% (control) to 68.3% (adding 3 μM **2a**) and 78.5% (adding 2 μM **2b**) via a concentration-dependent way. However, when the cells were pre-incubated with antioxidants, the mitochondrial membrane potential can be prevented from collapse in spite of the presence of **2a** or **2b** (Fig. 5a,b). With 0.075 mM LA pre-incubation, only approximate 12% of cells underwent the decrease of mitochondrial membrane potential, which were even less than that of control. These alterations can also be monitored by Rh123, which stains mitochondria specifically depending on membrane potential^38^. Although the intensity of Rh123 drop obviously in the presence of **2a** or **2b**, it could be recovered to some extent after pre-adding 0.075 mM LA (Fig. 5c). Furthermore, as shown in Fig. 5d, the relative content of cytochrome *c* in cells pre-incubated with 0.075 mM LA was similar with that of control, whereas the quantity was approximately six times (with 3 μM **2a**) or five times (with 2 μM **2b**) more than that of control. It was believed that the production of cytochrome *c* could be deterred effectively with the addition of 0.075 mM LA.

### The effect of antioxidants in cell death

**Figure 6:**
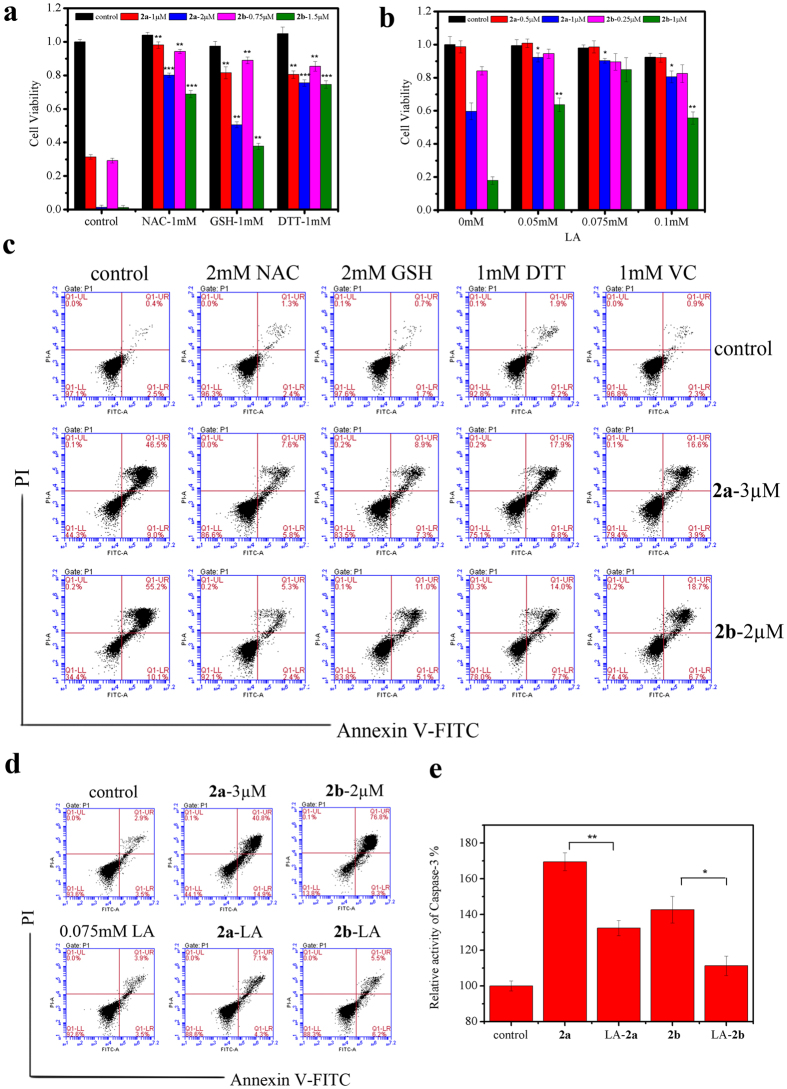
The effect of antioxidants on cell death. (**a**) Assessment of cell viability with the addition of NAC, GSH or DTT. HL-60 cells were incubated with **2a** or **2b** in the presence or absence of 1 mM NAC, 1 mM GSH, 1 mM DTT for 24 h and data are expressed as the mean ± SD of three independent samples. (**b**) The protective effect for cell viability with the different concentrations of LA. HL-60 cells were incubated with **2a** or **2b** in the presence or absence of LA with different concentrations for 24 h and the data are expressed as the mean ± SD of three independent samples. (**c**) Assessing the proportion of apoptotic cells in the presence of NAC, GSH, DTT or VC. HL-60 cells were treated with 3 μM **2a** or 2 μM **2b** and antioxidants for 24 h, followed by Annexin V-FITC/PI double staining. (**d**) Determination of the apoptotic cells percentage in the presence of LA. HL-60 cells were treated with 3 μM **2a** or 2 μM **2b** and 0.075 mM LA for 24 h, followed by Annexin V-FITC/PI double staining. (**e**) The increase of Caspase-3 activity. HL-60 cells were treated with 3 μM **2a** or 2 μM **2b** and 0.075 mM LA for 24 h and Caspase-3 activity in the cell extracts was determined by a colorimetric assay. *P < 0.05, **P < 0.01 and ***P < 0.001 vs the control group.

If **2a** and **2b** mainly aroused the oxidative stress, we speculated that the existence of antioxidants could maintain cells viable. In order to prove it, some related experiments with antioxidants were carried out continuously. As shown in Fig. 6a,b, the addition of antioxidants protected the majority of cells in the presence of low concentration drugs. In detail, after adding 1 mM NAC or 1 mM DTT, cell viability with low concentrations of drugs had a dramatic increase from about 0.3 to above 0.8, and that with high concentrations of drugs was from about 0 to above 0.7. The effect of 1 mM GSH was apparent for low concentration drugs but not obvious for high concentration drugs, while 0.075 mM LA can keep more than 80% cells live even exposed to the high concentration drugs. What’s more, the cell viability increased by an antioxidants concentration-dependent way (data not shown). Antioxidants with appropriate concentration were also used to study the effect on apoptosis rate. The results in Fig. 6c,d demonstrated that the apoptosis rates can be declined from 55.7% (**2a**) and 86.1% (**2b**) to 11.4% (**2a**) and 11.7% (**2b**) respectively with 0.075 mM LA pre-incubating, equal to that of control. Moreover, 2 mM NAC can also protect cells against apoptosis, which increased the proportion of normal cells from 44.3% (**2a**) and 34.4% (**2b**) to 86.6% (**2a**) and 92.1% (**2b**). Although 2 mM GSH and 1 mM DTT or VC were not so effective as NAC or LA, they can also decrease the apoptosis rates to some extent. What’s more, the activity of Caspase-3 indeed enhanced in the presence of only **2a** or **2b**, and the alteration could be deterred by 0.075 mM LA (Fig. 6e).

## Discussion

In order to develop organic arsenical drugs, we designed and synthesized a series of As (III)-containing Schiff bases with dual functional groups: As (III) and imine as well as two aromatic rings. It has been known that As (III) group in the molecules have high affinity with sulfhydryl to present inhibitory activities to sulfhydryl-containing proteins or enzymes^1^,^39^. The nitrogen with lone pair electrons in imine group displays basicity and nucleophilicity in biological system. Hydrophobic interaction may cause the binding of two hydrophobic aromatic rings with active sites in proteins and hence affect their conformation and bioactivities. Oxygen-containing substitutes at the aromatic rings and imine group are H-bond acceptors, which are able to impact H-bond network in protein and DNA. Therefore, a few As (III)-containing Schiff bases with Oxygen-containing substitutes at the aromatic rings have been synthesized.

From the results of cytotoxicity, it has been shown that organic arsenicals exhibit much higher inhibitory activity for cancer cells, especially for HL-60 cells, comparing with inorganic arsenicals. The *IC*_50_ values of **3a** and **4a** are both beyond 10 μM, which indicates that indole structure and anthracene ring structure may not be effective structures in this kind of Schiff base-compounds containing arsenic. On the contrary, the structure of salicylic aldehyde and vanillin are beneficial to biological activity. Taking all the data into account, it is concluded that **2a** and **2b** selectively and effectively inhibit the proliferation of HL-60 cells.

Considering the insensitivity of cancer cells to death, apoptosis-based strategies attract much attention in cancer research^40^. Arsenic compounds have been known to activate apoptosis signaling in numerous types of cell lines^32^,^33^. Moreover, the hallmarks of apoptosis include nuclear shrinkage, chromatin condensation, and apoptotic body formation^41^. As shown above, both **2a** and **2b** can trigger the apoptosis in HL-60 cells. Some reports have demonstrated that arsenic compounds induce the apoptosis by the way of the increase of ROS^1^,^3^,^23^,^34^. When ROS are generated excessively, including superoxide anion (O_2_^·−^), hydrogen peroxide (H_2_O_2_) hydroxyl radical (^·^OH), singlet oxygen (^1^O_2_), and perhydroxyl radical (HO^2·^) etc, it is involved in oxidative damage to lipids, proteins and DNA in cells^42^. The addition of **2a** or **2b** indeed increases the level of intracellular ROS and decreases the GSH content. It is known that arsenic can affect intracellular redox state through the higher affinity with the sulfydryl^37^. It was found that arsenic could target some enzymes, such as thioredoxin reductase, so the cells could only be protected by some individual antioxidants^15^. However, we found that the additional ROS can be eliminated by several general antioxidants, which were independent on the species of antioxidants. Furthermore, the loss of intracellular GSH can be recovered with the pretreatment of LA. It illustrates that **2a** and **2b** mainly led to the oxidative stress rather than targeting special proteins in HL-60 cells.

The mitochondrion is a major intracellular source of ROS, which is also the target of high ROS exposure with deleterious consequences, such as altering the mitochondrial membrane potential^43^. GSH serves as a sensitive marker of oxidative stress and it plays an important role in maintaining the integrity of mitochondria and cell membranes^44^. When the level of GSH decreased, especially mitochondrial GSH, it can induce the loss of mitochondrial membrane potential^36^,^37^. Besides that **2a** and **2b** can increase the production of ROS and depletion of GSH, they can also destroy the mitochondrial membrane potential. The mitochondria undergo major changes in membrane integrity when exposed to arsenicals, which leads to disruption of the mitochondrial membrane potential^37^,^45^. However, these alterations can also be recovered by the addition of LA. Based on these results, we put forward the point that the oxidative stress led to further damage to the mitochondrial membrane integrity. In addition, mitochondria are involved in the so-called intrinsic pathway of apoptosis. They release soluble proteins from the intermembrane space to initiate caspase activation in the cytosol, such as cytochrome *c*, which modulates the sensitivity to cell death signals^46^,^47^. The experimental results was in accordance with the observation above.

In addition, antioxidants with appropriate concentration can prevent the cells exposed to **2a** or **2b** from apoptosis. During the stage of apoptosis, the loss of the mitochondrial membrane can trigger the proteins release from mitochondria, which subsequently activates related catabolic proteases and nucleases^48^,^49^. Caspase-3 is included in caspases, a family of cysteine proteases mediating apoptosis by proteolysis of specific substrates, which is considered to be a primary executioner of apoptosis^50^,^51^. In our experiment, the activity of Caspase-3 enhanced by compound **2a** or **2b** could be weakened by LA. Considering along with the release of cytochrome *c*, it was believed that Caspase-3 was activated and apoptosis was induced. Taking all data into account, it was concluded that HL-60 cells underwent apoptosis and oxidative stress induced by **2a** or **2b** was the main cause.

In conclusion, this work has shed light on the antitumor activity and mechanism of several newly synthesized organic arsenicals *in vitro*. It is illustrated that **2a** and **2b** compounds show the best inhibitory activity for three different kinds of cancer cell lines, especially for HL-60 cells, which is far better than that of inorganic arsenicals, NaAsO_2_ and As_2_O_3_. It is found that both **2a** and **2b** have important influences on several critical points in apoptosis, including the burst of ROS, the depletion of GSH, collapse of mitochondrial membrane potential, release of cytochrome *c* and activation of Caspase-3. However, all the changes can be prevented by some antioxidants, particularly by lipoic acid. The results demonstrate that the apoptosis induced by **2a** or **2b** is mainly mediated by oxidative stress. This work provides deep insights into the design of organic arsenicals and the arsenic-related anticancer mechanism, which will attract much attention to the organic arsenicals as potential anticancer drugs.

## Materials and Methods

### Chemicals

*p*-Arsanilic acid, salicylaldehyde, vanillin, indole-3-mathanal, 9-anthraldehyde and aniline came from Shanghai Chemical Reagent Co. LTD in China. *p*-Arsanilic was purified via recrystallization in distilled water and salicylaldehyde was purified by reduced pressure distillation before use. RPMI 1640 Medium, Dulbecco’s modified Eagle Medium (DMEM) and Fetal bovine serum were purchased from GIBCO (Grand Island, USA). Rh123, Dimethyl sulfoxide (DMSO), N-acetyl-L-cysteine (NAC), reduced glutathione (GSH), DL-Dithiothreitol (DTT), L-Ascorbic acid (VC), (±)-α-Lipoic acid (LA) and 2′,7′-dichlorfluorescein diacetate (DCFH-DA) were obtained from Sigma-Aldrich (St. Louis, USA). 3-(4,5-dimethylthiazol-2-yl)-2,5-diphenyltetrazolium bromide (MTT) was obtained from Amresco (Solon, USA). NaAsO_2_ was purchased from Amresco (Solon, USA) and As_2_O_3_ was borrowed from Chemistry Experiment Teaching Center of Wuhan University. Mitochondria Staining Kit (JC-1) and Annexin V-FITC/PI apoptosis kit were purchased from MultiSciences (Hangzhou, China). Human Cyt *C* ELISA Kit was obtained from Elbio (Shanghai, China). Red Blood Cell Lysis Buffer, GSH and GSSG Assay Kit and the Activity of Caspase-3 Assay Kit were purchased from Beyotime (Shanghai, China). Other common chemicals were of analytical reagent grade from Wuhan Shenshi Chemical Reagent Co. LTD in China and used without further purification.

### Synthesis of As(III)-containing Schiff bases and 2-((phenylimino)methyl)phenol

Synthetic route of As (III)-containing Schiff bases was shown in Fig. 1. *p*-Aminophenylarsine oxide (**1**) can be produced from *p*-Arsanilic acid according to the general experimental procedure^52^. The reactant *p*-Aminophenylarsine oxide (10 mmol) or aniline (10 mmol) was dissolved in 60 mL of anhydrous ethanol placed into 250 mL of three-necked, round-bottomed flask equipped with a reflux condenser, a thermometer, a drop funnel and a stir bar. In a separate flask, equivalent another reactant aromatic aldehyde (10 mmol) was dissolved in 60 mL of anhydrous ethanol, then transferred by dropwise to the reaction mixture via the drop funnel under stirring. An additional 30 mL of ethanol was used to effect quantitative transfer of the aromatic aldehyde (10 mmol) into the reaction mixture. The mixture was refluxed and stirred for 2–6 h under nitrogen. At the end of reaction, 10 mmol of sodium hydroxide powder were added to drive the reaction towards the product side, and then the mixture was concentrated under reduced pressure. The resulting mixture was poured in distilled water, filtered and dried under vacuum. The crude product was recrystallized from ethanol/H_2_O or methanol/H_2_O.

### Characterization of synthetic organic arsenicals

Partial elemental analyses were performed on a Vario EL III CHNOS elemental analyzer. Infrared spectra were recorded on a Nicolet 380 FT-IR Spectrometer using KBr plates (4,000–400 cm^−1^). NMR spectra were obtained with a Varian Mercury VX300 spectrometer at 300 MHz using TMS as internal reference. MS were recorded on a Brucker Daltonics APE XII 47e and VG707VHF mass spectrometer.

2-((phenylimino) methyl) phenol (**1a**): Yellow powder. ^1^H NMR (300 MHz, DMSO-d_6_): 6.87–7.64 (m, 9H); 8.93 (S, 1H). ESI-MS, m/z: 198 [M + H]^+^.

2-(((4-(oxoarsanyl) phenyl) imino)methyl)phenol (**2a**): Yellow powder. Mp: 123–125 °C. Elemental analysis calculated for C_13_H_10_AsNO_2_: C, 54.38; H, 3.51; As, 26.09; N, 4.88; O, 11.14. Found: C, 54.32; H, 3.50; N, 4.91. IR (KBr): 3563 cm^−1^ (s, stretching of O-H); 1616 cm^−1^ (vs, stretching of CH=N); 1588, 1488, 1450 cm^−1^ (s, stretching of benzene cycle); 1083 cm^−1^ (s, stretching of ph-As). ^1^H NMR (300 MHz, CH_3_OH-d_4_): 4.85 (s,1 H); 6.87–7.75 (m, 8 H); 8.81 (S, 1 H). ^13^C NMR (100 MHz, CH_3_OH-d_4_): 117.2, 120.1, 121.4, 121.9, 122.1 (5C); 127.3, 132.5, 133.2, 133.7, 134.2 (5C); 149.3(1C); 161.8, 162.1(2C). ESI-MS, m/z: 288 [M + H]^+^.

2-methoxy-4-(((4-(oxoarsanyl)phenyl)imino)methyl)phenol (**2b**): Pale yellow and needle-shaped minicrystal. Mp: 102–103.5 °C. Elemental analysis calculated for C_14_H_12_AsNO_3_: C, 53.02; H, 3.81; As, 23.62; N, 4.42; O, 15.13. Found: C, 52.96; H, 3.78; N, 4.51. IR (KBr): 3316 cm^−1^ (vs, stretching of O-H); 1621cm^−1^ (vs, stretching of CH=N); 1593, 1500, 1423 cm^−1^ (s, stretching of benzene cycle); 1086 cm^−1^ (s, stretching of ph-As). ^1^H NMR (300 MHz, CH_3_OH-d_4_): 4.09 (s, 3H); 5.26 (s, 1H); 6.726 (d, 4 H); 7.331(m, 3 H); 8.401 (s, 1 H). ^13^C NMR (100 MHz, CH_3_OH-d_4_): 58.995(1C), 117.355, 117.760, 119.962, 120.749, 121.768, 122.095 (6C), 130.469, 131.364, 132.586, 133.325, 134.212, 147.326 (6C), 149.333 (1C). ESI-MS, m/z: 318 [M + H]^+^.

1-(1H-indol-3-yl)-N-(4-(oxoarsanyl) phenyl) methanimine (**3a**): Pale yellow powder. Mp: 196–198 °C. Elemental analysis calculated for C_15_H_11_AsN_2_O: C, 58.08; H, 3.57; As, 24.15; N, 9.03; O, 5.16. Found: C, 58.06; H, 3.56; N, 9.05. IR (KBr): 3492 cm^−1^ (m, stretching of N-H); 3069 cm^−1^ (s, stretching of ph-H); 1621 cm^−1^(vs, stretching of CH=N); 1587, 1504, 1479 cm^−1^ (s, stretching of benzene cycle); 1084 cm^−1^ (m, stretching of ph-As). ^1^H NMR (300 MHz, DMSO-d_6_): 7.11–7.72(m, 9 H); 8.22 (s, 1 H); 9.97(s, 1 H). ^13^C NMR (100 MHz, DMSO-d_6_): 113.1(2C);118.8(1C); 121.5 (2C); 122.8 (2C); 124.2 (2C); 124.8 (1C); 137.7 (1C); 139.2 (2C); 185.7 (2C). ESI-MS, m/z: 311 [M + H]^+^.

1-(anthracen-9-yl)-N-(4-(oxoarsanyl) phenyl) methanimine (**4a**): White powder. Mp: 91–92 °C. Elemental analysis calculated for C_21_H_14_AsNO: C, 67.94; H, 3.80; As, 20.18; N, 3.77; O, 4.31. Found: C, 67.92; H, 3.79; N, 3.79. IR (KBr): 3036 cm^−1^ (s, stretching of ph-H); 1610 cm^−1^(s, stretching of CH=N); 1594, 1501, 1413 cm^−1^ (s, stretching of benzene cycle); 1078 cm^−1^ (m, stretching of ph-As). ^1^H NMR (300 MHz, DMSO-d_6_): 7.36–9.04 (m, 12 H); 9.78 (s, 1 H); 11.46 (s, 1 H). ^13^C NMR (100 MHz, DMSO-d_6_): 121.9, 124.2, 125.1, 125.6, 126.5, 127.4, 128.2, 130.0, 130.1, 130.8, 131.3, 131.5, 132.1, 136.0(24C), 194.9(1C). ESI-MS, m/z: 371[M + H]^+^.

### Cell cultures

HL-60 cells were obtained from College of Life Sciences at Wuhan University. SGC 7901 cells and GES-1 cells were obtained from Prof. Xiang Zhou’s group. MCF-7 cells and HEK293 cells were obtained from Prof. Dai-Wen Pang’s group and Prof. Xian-Zheng Zhang’s group, respectively. NIH3T3 cells were obtained from China Center for Type Culture Collection at Wuhan University. HL-60 cells were cultured in RPMI 1640 Medium containing 10% FBS and 1% penicillin/streptomycin solution. SGC 7901 cells, MCF-7 cells, GES-1 cells, HEK293 cells and NIH3T3 cells were cultured in DMEM containing 10% FBS, 2 mM glutamine, 0.1 μM sodium bicarbonate and 1% penicillin/streptomycin solution. They were all maintained in an atmosphere of 5% CO_2_ at 37 °C.

### Assessment of cell viability

1 × 10^4^ HL-60 cells and drugs with different concentrations in a final volume of 80 μL were incubated in a 96-well plate. The drugs were dissolved in DMSO and the concentrations of DMSO were under 0.1% (*V*/*V*). Medium only, cells only and cells treated with DMSO alone were taken into account as controls. After 24 h, 20 μL MTT (5 mg/mL) was added to each well. After 4 h, 100 μL 10% SDS solution was added to each well and the plate was placed in the dark at 37 °C overnight. For other cells, after incubation with drugs for 24 h, the medium was removed and the cells were washed once with PBS. Then, 200 μL medium containing 20 μL MTT (5 mg/mL) was added to each well. After 4 h, the medium was removed and 150 μL DMSO was added to each well and the plate was placed in the dark at 37 °C for 15 min under shaking. The absorbance was measured at 570 nm with microplate reader (BioTek, USA).

5 × 10^4^ HL-60 cells and different antioxidants with different concentrations were incubated in a 48-well plate for 4 h. Then **2a** or **2b** with different concentrations was added into the sample well. Cells only and cells treated with antioxidants alone were taken into account as controls. After 24 h, the cells were stained with trypan blue (0.4%, w/v) and the number of live (non-stained) and dead (stained) cells were obtained using the microscope.

### Determination of the intracellular ROS level

HL-60 cells treated with 2 μM **2a** or 1.5 μM **2b** were plated in 12-well plates. After 24 h, each sample of cells was collected and washed once with PBS. The cells were incubated with 300 μL FBS-free medium containing DCFH-DA (1 μM) for 30 min in dark at 37 °C. Data were obtained and analyzed using a C6 flow cytometer (BD Biosciences, USA)^27^.

In laser scanning confocal experiment, the medium was removed after 24 h incubation. 1 mL FBS-free medium with DCFH-DA (10 μM) was added to each sample. Then continue the incubation for 30 min in dark at 37 °C. The samples were visualized and photographed using a C1-si Laser Scanning Confocal Microscope (Nikon, Japan).

### Assessment of the total glutathione and GSH level

The cells were cultured in a 6-well plate and treated with 2 μM **2a** or 1.5 μM **2b**. After 24 h, the cells were collected and washed twice with PBS. The protein content was quantified by the use of Bradford procedure. Then each sample was treated according to the manufacturer’s instructions (beyotime, Shanghai, China). The absorbance was measured every 5 minutes at 405 nm with microplate reader.

### Identification of mitochondrial membrane potential

HL-60 cells treated with **2a** (1.5 μM and 3 μM) or **2b** (1 μM and 2 μM) were plated in 12-well plates. After 24 h, cells were collected and washed once with PBS. Then, the cells were handled with JC-1 according to the manufacturer’s instructions (Multi Sciences BiotechCo., Ltd, Hangzhou, China) or Rh123. Data were obtained and analyzed using a C6 flow cytometer (BD Biosciences, USA).

### Apoptosis detection with Annexin V-FITC and PI staining

HL-60 cells were treated with **2a** (1.5 μM and 3 μM) or **2b** (1 μM and 2 μM) for 24 h in a 6-well plate. After 24 h, cells were collected and washed once with PBS. Then, the cells were handled with Annexin V-FITC and PI according to the manufacturer’s instructions (Multi Science sBiotechCo., Ltd, Hangzhou, China). Data were obtained and analyzed using a C6 flow cytometer (BD Biosciences, USA).

### Assessment of the released cytochrome *c* level

HL-60 cells with 3 μM **2a** or 2 μM **2b** were incubated in a 12-well plate for 24 h. The cells were sacrificed and dealt with ELISA Kit (Meilian BiotechCo., Ltd, Shanghai, China) according to the manufacturer’s instructions. The absorbance was measured at 450 nm with microplate reader.

### Hoechst 33342 staining

HL-60 cells treated with **2a** (1 μM and 2 μM) or **2b** (0.75 μM and 1.5 μM) were plated in 12-well plates, and the medium was removed after 24 h, followed by the addition of 5 μg.mL^−1^ Hoechst 33342. After being incubated for 30 min in dark at 37 °C, the samples were visualized and photographed using a C1-si Laser Scanning Confocal Microscope (Nikon, Japan)^27^.

### Measurement of caspase-3 activity

HL-60 cells were treated with 3 μM **2a** or 2 μM **2b** for 12 h in a 6-well plate. Each sample was collected and washed twice with PBS. Then the cells were incubated with moderate lysis buffer from the kit on ice. The cell extract was handled on the basis of manufacturer’s instructions (beyotime, Shanghai, China). After 3 h, the absorbance at 405 nm was measured using a microplate reader.

### The protective experiments using protective agents

In protective trial, the cells were pre-incubated with the protective agents for 4 h and other operations were in accordance with the above.

### Assessment of effect on normal leukocyte

Normal human fresh peripheral blood with EDTA anticoagulant was dealt with Red Blood Cell Lysis Buffer according to manufacturer’s instructions (Beyotime, Shanghai, China) to obtain the normal leukocyte. After the cell viability of leukocyte determined by trypan blue was confirmed above 95%, 2 × 10^4^ leukocyte cells were incubated with 3.5 μM **2a** (5 × *IC*_50_), 2.5 μM **2b** (5 × *IC*_50_), or 15 μM As_2_O_3_ (2.5 × *IC*_50_) for 6 h, 12 h or 24 h in RPMI 1640 at room temperature. Then, the apoptosis rate using Annexin V-FITC/PI staining and cell viability using MTT method were tested respectively. There are 8 independent normal human fresh peripheral blood samples in all to be tested. This study was carried out in accordance with relevant guidelines and regulations of Wuhan University and the experiment protocol was approved by Zhongnan Hospital of Wuhan University. Additionally, the informed consent was obtained from all subjects.

### Statistics

All experiments were performed at least three replicates. The data were presented as mean ± SD. The independent Student’s T test was used to compare the means of two independent groups. Significance was set at P < 0.05.

## Additional Information

**How to cite this article**: Fan, X.-Y. *et al*. Oxidative stress-mediated intrinsic apoptosis in human promyelocytic leukemia HL-60 cells induced by organic arsenicals. *Sci. Rep.* **6**, 29865; doi: 10.1038/srep29865 (2016).

## Supplementary Material

Supplementary Information

## Figures and Tables

**Table 1:** Chemical structure of synthesized arsenic (III)-containing Schiff base derivatives and their half-inhibitory concentrations *IC*_50_ for four different types of cell lines^a^.

Compound	cell lines (*IC*_5*0*_)			
	HL-60	MCF-7	SGC7901	GES-1
NaAsO_2_	10.55 ± 3.06	31.66 ± 0.67	58.95 ± 11.84	42.6 ± 11.62
As_2_O_3_	6.44 ± 1.62	30.60 ± 2.80	41.35 ± 2.70	49.08 ± 2.89
**1a**	>100	>100	>100	>100
**2a**	0.77 ± 0.1	2.53 ± 0.41	4.03 ± 0.89	5.96 ± 1.23
**2b**	0.51 ± 0.1	2.81 ± 1.34	2.12 ± 0.48	1.57 ± 0.26
**3a**	13.94 ± 0.47	69.00 ± 9.01	155.03 ± 7.85	83.3 ± 4.26
**4a**	15.89 ± 0.56	11.29 ± 3.40	7.63 ± 3.02	8.72 ± 3.76

^a^The data (*IC*_50_) are expressed as the mean ± SD of three independent experiments and the unit is μmol·L^−1^.

## References

[b1] Shen S., Li X.-F., Cullen W. R., Weinfeld M. & Le X. C. Arsenic binding to proteins. Chem Rev 113, 7769–7792 (2013). 23808632 10.1021/cr300015c PMC3797521

[b2] Jomova K. . Arsenic: toxicity, oxidative stress and human disease. J Appl Toxicol 31, 95–107 (2011). 21321970 10.1002/jat.1649

[b3] Chatterjee S., Sarkar S. & Bhattacharya S. Toxic Metals and Autophagy. Chem Res Toxicol 27, 1887–1900 (2014). 25310621 10.1021/tx500264s

[b4] Ngu T. T. & Stillman M. J. Arsenic binding to human metallothionein. J Am Chem Soc 128, 12473–12483 (2006). 16984198 10.1021/ja062914c

[b5] Liu S. . Arsenic Induced Overexpression of Inflammatory Cytokines Based on the Human Urothelial Cell Model *in Vitro* and Urinary Secretion of Individuals Chronically Exposed to Arsenic. Chem Res Toxicol 27, 1934–1942 (2014). 25257954 10.1021/tx5002783

[b6] Lo-Coco F. . Retinoic acid and arsenic trioxide for acute promyelocytic leukemia. New Engl J Med 369, 112–121 (2013). 10.1056/NEJMoa1300874 23841729

[b7] Coombs C. C., Tavakkoli M. & Tallman M. S. Acute promyelocytic leukemia: where did we start, where are we now, and the future. Blood Cancer J 5, e304 (2015). 25885425 10.1038/bcj.2015.25 PMC4450325

[b8] Smith B. R., Eastman C. M. & Njardarson J. T. Beyond C, H, O and N! Analysis of the Elemental Composition of US FDA Approved Drug Architectures. J Med Chem 57, 9764–9773 (2014). 25255063 10.1021/jm501105n

[b9] Jia P. G. & Huang X. Arsenic trioxide induces multiple myeloma cell apoptosis via disruption of mitochondrial transmembrane potentials and activation of caspace-3. Chin Med J 114, 19–24 (2001). 11779429

[b10] Evens A. M., Tallman M. S. & Gartenhaus R. B. The potential of arsenic trioxide in the treatment of malignant disease: past, present, and future. Leuk Res 28, 891–900 (2004). 15234563 10.1016/j.leukres.2004.01.011

[b11] Eguchi R. . Arsenic trioxide induces apoptosis through JNK and ERK in human mesothelioma cells. J Cell Physiol 226, 762–768 (2011). 20799280 10.1002/jcp.22397

[b12] Oketani M. . Inhibition by arsenic trioxide of human hepatoma cell growth. Cancer Lett 183, 147–153 (2002). 12065089 10.1016/s0304-3835(01)00800-x

[b13] Waxman S. & Anderson K. C. History of the development of arsenic derivatives in cancer therapy. Oncologist 6 Suppl 2, 3–10 (2001). 11331434 10.1634/theoncologist.6-suppl_2-3

[b14] Dilda P. J. & Hogg P. J. Arsenical-based cancer drugs. Cancer Treat Rev 33, 542–564 (2007). 17624680 10.1016/j.ctrv.2007.05.001

[b15] Liu Y. . Dithiaarsanes inducing oxidative stress-mediated apoptosis in HL-60 cells by selectively targeting the thioredoxin reductase. J Med Chem 57, 5203–5211 (2014). 24867309 10.1021/jm500221p

[b16] Thompson R. C. & Smith D. C. Evaluation of the treatment of early syphilis with arsphenamine and heavy metal. Am J Syph Gonorrhea Vener Dis 34, 410–419 (1950). 14771369

[b17] Galluzzi L. . Molecular definitions of cell death subroutines: recommendations of the Nomenclature Committee on Cell Death 2012. Cell Death Differ 19, 107–120 (2011). 21760595 10.1038/cdd.2011.96 PMC3252826

[b18] Reed J. C. Mechanisms of apoptosis. Am J Pathol 157, 1415–1430 (2000). 11073801 10.1016/S0002-9440(10)64779-7 PMC1885741

[b19] Strasser A., Oconnor L. & Dixit V. M. Apoptosis signaling. Annu Rev Biochem 69, 217–245 (2000). 10966458 10.1146/annurev.biochem.69.1.217

[b20] Callista Y., Wen Y. & Siegfried H. The intrinsic apoptosis pathway mediates the pro-longevity response to mitochondrial ROS in C. elegans. Cell 157, 897–909 (2014). 24813612 10.1016/j.cell.2014.02.055 PMC4454526

[b21] Chipuk J. & Green D. Dissecting p53-dependent apoptosis. Cell Death Differ 13, 994–1002 (2006). 16543937 10.1038/sj.cdd.4401908

[b22] Kang J. M. . Effect of aging on gastric mucosal defense mechanisms: ROS, apoptosis, angiogenesis, and sensory neurons. Am J Physiol Gastrointest Liver Physiol 299, G1147–G1153 (2010). 20724528 10.1152/ajpgi.00218.2010

[b23] Zamora P. L., Rockenbauer A. & Villamena F. A. Radical Model of Arsenic (III) Toxicity: Theoretical and EPR Spin Trapping Studies. Chem Res Toxicol 27, 765–774 (2014). 24754521 10.1021/tx4004227

[b24] Slyemi D. & Bonnefoy V. How prokaryotes deal with arsenic. Environ Microbiol Rep 4, 571–586 (2012). 23760928 10.1111/j.1758-2229.2011.00300.x

[b25] Indran I. R., Hande M. P. & Pervaiz S. hTERT overexpression alleviates intracellular ROS production, improves mitochondrial function, and inhibits ROS-mediated apoptosis in cancer cells. Cancer Res 71, 266–276 (2011). 21071633 10.1158/0008-5472.CAN-10-1588

[b26] Navarro R., Busnadiego I., Ruiz-Larrea M. B. & Ruiz-Sanz J. I. Superoxide Anions Are Involved in Doxorubicin-Induced ERK Activation in Hepatocyte Cultures. Ann N Y Acad Sci 1090, 419–428 (2006). 17384286 10.1196/annals.1378.045

[b27] Duan D., Zhang B., Yao J., Liu Y. & Fang J. Shikonin targets cytosolic thioredoxin reductase to induce ROS-mediated apoptosis in human promyelocytic leukemia HL-60 cells. Free Radic Biol Med 70, 182–193 (2014). 24583460 10.1016/j.freeradbiomed.2014.02.016

[b28] Simon H.-U., Haj-Yehia A. & Levi-Schaffer F. Role of reactive oxygen species (ROS) in apoptosis induction. Apoptosis 5, 415–418 (2000). 11256882 10.1023/a:1009616228304

[b29] da Silva C. M. . Schiff bases: A short review of their antimicrobial activities. J Adv Res 2, 1–8 (2011).

[b30] Yanhua Z. . Arsenic trioxide (As(2)O(3)) induces apoptosis through activation of Bax in hematopoietic cells. Oncogene 24, 3339–3347 (2005). 15735709 10.1038/sj.onc.1208484

[b31] Rocha R. A. . Arsenic and fluoride induce neural progenitor cell apoptosis. Toxicol Lett 203, 237–244 (2011). 21439358 10.1016/j.toxlet.2011.03.023

[b32] Zhang Y. . Arsenic trioxide induced apoptosis in retinoblastoma cells by abnormal expression of microRNA-376a. Neoplasma 60, 247–253 (2013). 23373993 10.4149/neo_2013_033

[b33] Yen Y. P. . Arsenic induces apoptosis in myoblasts through a reactive oxygen species-induced endoplasmic reticulum stress and mitochondrial dysfunction pathway. Arch Toxicol 86, 923–933 (2012). 22622864 10.1007/s00204-012-0864-9

[b34] Cheng Y. . Neuroprotective effect of resveratrol on arsenic trioxide–induced oxidative stress in feline brain. Hum Exp Toxicol 33, 737–747 (2014). 24107457 10.1177/0960327113506235

[b35] Piga R., Saito Y., Yoshida Y. & Niki E. Cytotoxic effects of various stressors on PC12 cells: involvement of oxidative stress and effect of antioxidants. Neurotoxicology 28, 67–75 (2007). 16942797 10.1016/j.neuro.2006.07.006

[b36] Li J.-j. . Role of oxidative stress in the apoptosis of hepatocellular carcinoma induced by combination of arsenic trioxide and ascorbic acid. Acta Pharmacol Sin 27, 1078–1084 (2006). 16867262 10.1111/j.1745-7254.2006.00345.x

[b37] Hu X.-M., Hirano T. & Oka K. Arsenic trioxide induces apoptosis in cells of MOLT-4 and its daunorubicin-resistant cell line via depletion of intracellular glutathione, disruption of mitochondrial membrane potential and activation of caspase-3. Cancer Chemother Pharmacol 52, 47–58 (2003). 12750841 10.1007/s00280-003-0629-5

[b38] Shapiro H. M. Membrane potential estimation by flow cytometry. Methods 21, 271–279 (2000). 10873481 10.1006/meth.2000.1007

[b39] Spuches A. M., Kruszyna H. G., Rich A. M. & Wilcox D. E. Thermodynamics of the As(III)-thiol interaction: arsenite and monomethylarsenite complexes with glutathione, dihydrolipoic acid, and other thiol ligands. Inorg Chem 44, 2964–2972 (2005). 15819584 10.1021/ic048694q

[b40] Gregory C. D. & Pound J. D. Microenvironmental influences of apoptosis *in vivo* and *in vitro*. Apoptosis 15, 1029–1049 (2010). 20237956 10.1007/s10495-010-0485-9

[b41] Giansanti V., Torriglia A. & Scovassi A. I. Conversation between apoptosis and autophagy: “Is it your turn or mine?”. Apoptosis 16, 321–333 (2011). 21404107 10.1007/s10495-011-0589-x

[b42] Chen C., Jiang X., Ren Y. & Zhang Z. Arsenic trioxide co-exposure potentiates benzo (a) pyrene genotoxicity by enhancing the oxidative stress in human lung adenocarcinoma cell. Biol Trace Elem Res 156, 338–349 (2013). 24061964 10.1007/s12011-013-9819-0

[b43] Circu M. L. & Aw T. Y. Reactive oxygen species, cellular redox systems, and apoptosis. Free Radic Biol Med 48, 749–762 (2010). 20045723 10.1016/j.freeradbiomed.2009.12.022 PMC2823977

[b44] Husain K., Whitworth C. & Rybak L. P. Time response of carboplatin-induced nephrotoxicity in rats. Pharmacol Res 50, 291–300 (2004). 15225673 10.1016/j.phrs.2004.04.001

[b45] Jia P. . Arsenic trioxide induces multiple myeloma cell apoptosis via disruption of mitochondrial transmembrane potentials and activation of caspase-3. Chin Med J 114, 19–24 (2001). 11779429

[b46] Skommer J., Brittain T. & Raychaudhuri S. Bcl-2 inhibits apoptosis by increasing the time-to-death and intrinsic cell-to-cell variations in the mitochondrial pathway of cell death. Apoptosis 15, 1223–1233 (2010). 20563668 10.1007/s10495-010-0515-7 PMC2948171

[b47] Martinou J.-C. & Youle R. J. Mitochondria in apoptosis: Bcl-2 family members and mitochondrial dynamics. Dev Cell 21, 92–101 (2011). 21763611 10.1016/j.devcel.2011.06.017 PMC3156409

[b48] Iguchi K., Hirano K. & Ishida R. Activation of caspase-3, proteolytic cleavage of DFF and no oligonucleosomal DNA fragmentation in apoptotic Molt-4 cells. J Biochem 131, 469–475 (2002). 11872177 10.1093/oxfordjournals.jbchem.a003123

[b49] Zhu X.-F. . Involvement of caspase-3 activation in squamocin-induced apoptosis in leukemia cell line HL-60. Life Sci 70, 1259–1269 (2002). 11883704 10.1016/s0024-3205(01)01501-6

[b50] Carambula S. F. . Caspase-3 is a pivotal mediator of apoptosis during regression of the ovarian corpus luteum. Endocrinology 143, 1495–1501 (2002). 11897708 10.1210/endo.143.4.8726

[b51] Brentnall M., Rodriguez-Menocal L., De Guevara R. L., Cepero E. & Boise L. H. Caspase-9, caspase-3 and caspase-7 have distinct roles during intrinsic apoptosis. BMC Cell Biol 14, 32 (2013). 23834359 10.1186/1471-2121-14-32 PMC3710246

[b52] Wilson P. . Organic arsenicals as efficient and highly specific linkers for protein/peptide-polymer conjugation. J Am Chem Soc 137, 4215–4222 (2015). 25794267 10.1021/jacs.5b01140

