# Supplementary material for: Oxidative stress-mediated intrinsic apoptosis in human promyelocytic leukemia HL-60 cells induced by organic arsenicals
Source: Sci Rep. 2016 Jul 19;6:29865. doi: 10.1038/srep29865 (PMC4949440; doi:10.1038/srep29865)
Supplement: Supplementary Information [file srep29865-s1.pdf]

Supporting Information

Oxidative stress-mediated intrinsic apoptosis in human promyelocytic leukemia HL-60 cells induced by organic arsenicals

Xiao-Yang Fan[a,c], Xin-You Chen[b,c], Yu-Jiao Liu[a], Hui-Min Zhong[a], Feng-Lei Jiang[a], Yi Liu[a,b,*]

Table S1. Cytotoxicity of compound **2a**, **2b** and inorganic arsenicals towards HEK293 and NIH3T3 cells[a].

Fig. S1 The effect of **2a**, **2b** and As_2O_3 on cell viability of leukocyte

Fig. S2 Flow cytometric analysis of **2a**, **2b** and As_2O_3 on apoptosis of leukocyte

Table S1. Cytotoxicity of compound **2a**, **2b** and inorganic arsenicals towards HEK293 and NIH3T3 cells[a].

Compound	cell lines (IC_{50})	
	HEK293	NIH3T3
**2a**	4.36±0.62	7.43±0.37
**2b**	2.29±0.34	2.72±0.64
As_2O_3	14.52±1.92	6.94±1.04
$NaAsO_2$	14.56±0.61	10.79±1.66

[a]The data (IC_{50}) are expressed as the mean ± SD of three independent experiments and the unit is $\mu mol \cdot L^{-1}$.

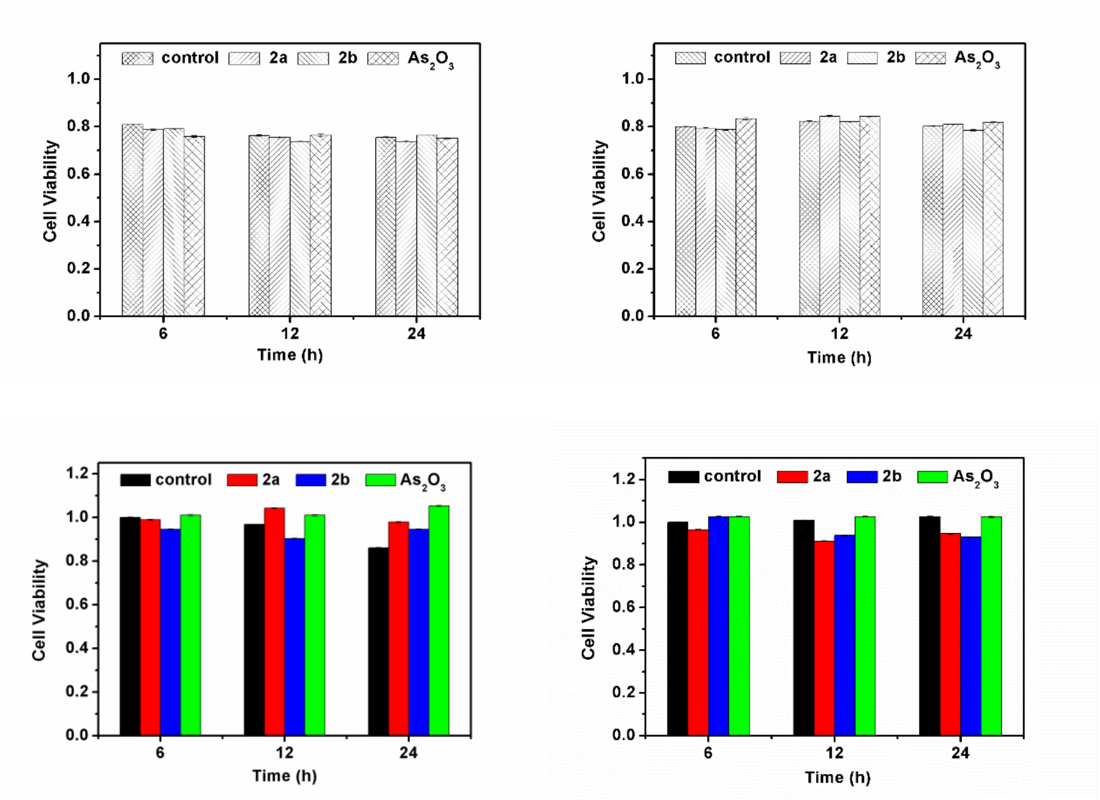

Fig. S1. The effect of 3.5 μM **2a**, 2.5 μM **2b** and 15 μM As_2O_3 on cell viability of leukocyte

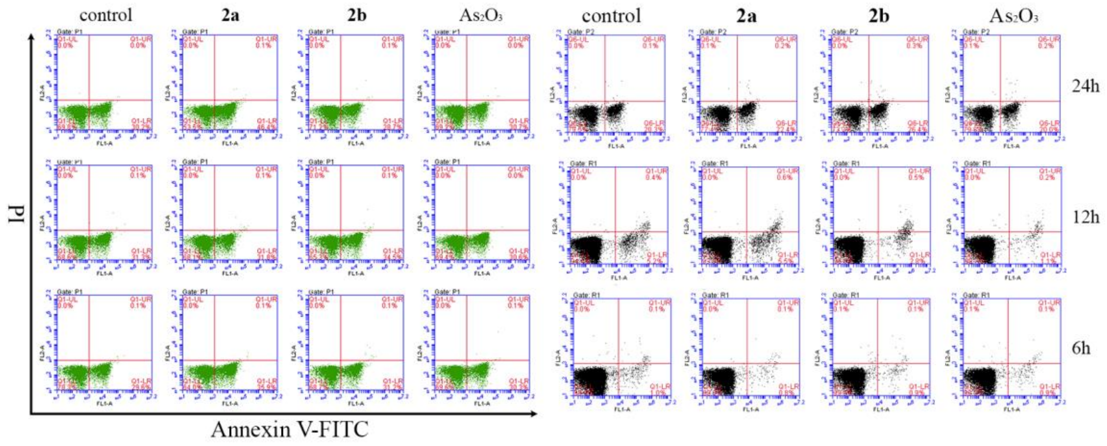

Fig. S2. Flow cytometric analysis of 3.5 µM **2a**, 2.5 µM **2b** and 15 µM As_2O_3 on apoptosis of leukocyte
